# Dosimetric effects of anatomical changes during fractionated photon radiation therapy in pancreatic cancer patients

**DOI:** 10.1002/acm2.12199

**Published:** 2017-10-04

**Authors:** Astrid van der Horst, Antonetta C. Houweling, Geertjan van Tienhoven, Jorrit Visser, Arjan Bel

**Affiliations:** ^1^ Department of Radiation Oncology Academic Medical Center University of Amsterdam Amsterdam the Netherlands

**Keywords:** anatomical changes, dose accumulation, fiducial markers, gastrointestinal gas, pancreatic cancer, radiotherapy

## Abstract

Pancreatic tumors show large interfractional position variation. In addition, changes in gastrointestinal gas volumes and body contour take place over the course of radiation therapy. We aimed to quantify the effect of these anatomical changes on target dose coverage, for the clinically used fiducial marker‐based patient position verification and, for comparison, also for simulated bony anatomy‐based position verification. Nine consecutive patients were included in this retrospective study. To enable fraction dose calculations on cone‐beam CT (CBCT), the planning CT was deformably registered to each CBCT (13–15 per patient); gas volumes visible on CBCT were copied to the deformed CT. Fraction doses were calculated for the clinically used 10 MV VMAT treatment plan (with for the planning target volume (PTV): D_98%_ = 95%), according to fiducial marker‐based and bony anatomy‐based image registrations. Dose distributions were rigidly summed to yield the accumulated dose. To evaluate target dose coverage, we defined an iCTV_+5 mm_ volume, i.e., the internal clinical target volume (iCTV) expanded with a 5 mm margin to account for remaining uncertainties including delineation uncertainties. We analyzed D_98%_, D_mean_, and D_2%_ for iCTV_+5 mm_ and PTV (i.e., iCTV plus 10 mm margin). We found that for fiducial marker‐based registration, differences between fraction doses and planned dose were minimal. For bony anatomy‐based registration, fraction doses differed considerably, resulting in large differences between planned and accumulated dose for some patients, up to a decrease in D_98%_ of the iCTV_+5 mm_ from 95.9% to 85.8%. Our study shows that fractionated photon irradiation of pancreatic tumors is robust against variations in body contour and gastrointestinal gas, with dose coverage only mildly affected. However, as a result of interfractional tumor position variations, target dose coverage can severely decline when using bony anatomy for patient position verification. Therefore, the use of intratumoral fiducial marker‐based daily position verification is essential in pancreatic cancer patients.

## 1 INTRODUCTION

For pancreatic cancer, radiation therapy is typically planned using a single (three‐ or four‐dimensional) computed tomography (CT) scan. Between acquiring this planning CT and the start of radiation therapy, as well as during radiation therapy, anatomical changes may take place, including interfractional target position variation and changes in body contour and gastrointestinal gas. Such anatomical changes will introduce differences between planned and delivered dose, possibly compromising target dose coverage. To assess the necessity of monitoring and/or compensating for anatomical changes over the course of radiation therapy, the robustness of treatment plans against these anatomical changes must be investigated.

The dosimetric effects of changes in body contour, e.g., due to weight change, have been studied for head and neck^1^, ^2^ and prostate cancer patients.^3^, ^4^ For tumor sites in the upper abdomen, including the pancreas, no studies were found.

Dosimetric studies into the effect of gastrointestinal gas changes are scarce. In five‐fraction stereotactic body radiation therapy (SBRT) in pancreatic cancer patients, variation in gastric gas volume substantially changed the dose to the planning target volume (PTV).^5^ However, when radiation therapy consists of more fractions, the effects of gastrointestinal gas on dose are more likely to average out. For intensity‐modulated radiation therapy (IMRT) plans, the dosimetric impact of gastrointestinal gas in pancreatic cancer patients was found to be limited to a few percent, even with large variations in gas volume;^6^ only weekly cone‐beam CT (CBCT) scans were used, though, preventing analysis of the full effect on accumulated target dose.

Interfractional position variation of the target relative to the bony anatomy can be considerable in pancreatic cancer patients;^7^, ^8^, ^9^ in an earlier study, we found in 13 patients a three‐dimensional vector displacement of >10 mm in 116 out of 300 fractions.^10^ Intratumoral fiducial markers may be used for daily position verification to mitigate this positional uncertainty. The dosimetric effects of interfractional position variation have been investigated in pancreatic cancer patients.^11^, ^12^, ^13^ However, in most of these studies, only three or five CBCT or CT scans per patient were used, which may not be representative for a 3‐week 15‐fraction treatment schedule.^11^, ^12^ One study (10 patients) used 107 daily CT scans obtained with an in‐room CT‐on‐rails to characterize anatomical changes and study the dosimetric advantages of online replanning, but did not quantify gastrointestinal gas changes.^13^

The aim of our study was to quantify for fractionated photon radiation therapy of pancreatic cancer patients the effect on target dose coverage as a result of changes in body contour, gastrointestinal gas, and tumor position. To do so, we accumulated fraction doses, calculated based on 13–15 daily CBCTs per patient, and analyzed target dose coverage.

## 2 METHODS

### 2.A Patient population

We included nine consecutive patients with (borderline) resectable pancreatic cancer in this retrospective treatment planning study. Inclusion criteria were: intratumoral fiducial markers visible on the planning CT (pCT) and daily CBCTs; patient contour visible over the extent of each CBCT. Implantation of fiducial markers is standard clinical care for (borderline) resectable pancreatic cancer patients in our institute; the fiducial markers (2–4 per patient) are used for daily online CBCT‐based position verification.^10^, ^14^

Patients were treated between July 2013 and October 2014 with a planned therapy scheme of 13 × 3.0 Gy (patient 7) or 15 × 2.4 Gy (all others; these patients participated in the PREOPANC trial^15^). The daily CBCT scans were obtained in free breathing prior to irradiation (Synergy system; Elekta Oncology Systems, Crawley, UK); a total of 132 daily CBCTs (13–15 per patient) were available.

### 2.B Target volumes and treatment planning

In our study we used the target volumes and treatment plans as used for these patients in the clinic. The target volumes had been delineated on the pCT, which was either a three‐dimensional (3D)‐CT (patient 1) or a 4D‐CT consisting of ten respiratory phase scans. The contoured gross target volume (GTV) included suspicious lymph nodes and fat infiltration and had been expanded to an internal GTV (iGTV) that encompassed the GTV on each respiratory phase scan. A margin of 5 mm for microscopic tumor extension had been applied to the iGTV to create the internal clinical target volume (iCTV).^16^ For patient 1, the CTV (i.e., the GTV plus a 5 mm margin) as defined on the 3D‐CT was used. Application of a 10 mm margin to the iCTV or CTV resulted in the PTV.

We used the 10 MV single‐arc volumetric modulated arc therapy (VMAT) plans as used in the clinic, for which the dose to 99% of the PTV had to be at least 95% of the prescribed dose (36 or 39 Gy), the mean kidney dose could not exceed 18 Gy and the mean liver dose had to be ≤30 Gy. With prescription doses of 36 and 39 Gy, constraints for spinal cord, stomach and bowel were irrelevant. Stomach and duodenum were delineated for the purpose of this study by one physician experienced in delineation in pancreatic cancer patients.

### 2.C CBCT dose calculations and dose accumulation

**Figure 1:**
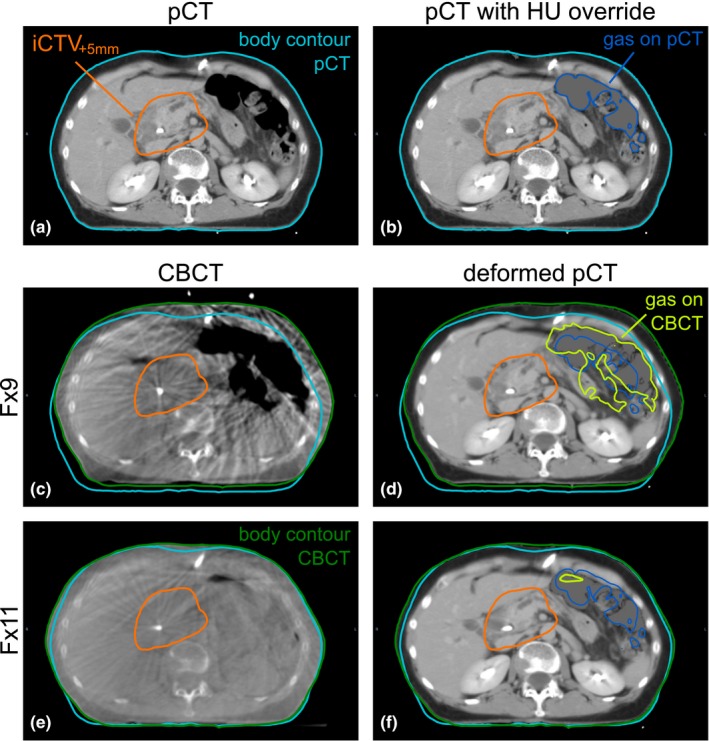
Anatomical changes. Example of anatomical changes in patient 8: (a) pCT, (b) pCT with HU override of 0 for gastrointestinal gas, (c) CBCT for fraction 9, (d) deformed CT for fraction 9, (e) CBCT for fraction 11, and (f) deformed CT for fraction 11. Images in (d and f) are for the fiducial marker‐based rigid registration to the pCT. Contours are shown for iCTV_+5 mm_ (orange), body contour in pCT (light blue), gas in pCT (dark blue), body contour in the CBCTs (green), and gas in the CBCTs (yellow). The gas volumes on CBCT have been copied to the deformed CT and given a density override of 0.01. Relevant gas volumes: (b) 237 cm^3^; (d) 503 cm^3^; (f) 21 cm^3^.

As CBCT Hounsfield units (HUs) are inaccurate, performing dose calculations directly on CBCT was not feasible. Therefore, using a method previously applied by our group,^17^, ^18^ we deformably registered the pCT [Fig. 1(a)] to each CBCT (Velocity, version 3.1, Varian Medical Systems, Palo Alto, CA, USA). To not have this registration be driven by the gastrointestinal gas volumes, we first applied an override to the gastrointestinal gas in the pCT: all voxels with a HU value <−150 were assigned a HU value of 0 [Fig. 1(b)]. For each CBCT, the registration of body contour, bony anatomy, fiducial markers, and clear organ boundaries (e.g., kidneys) was visually checked. After deformable registration of the pCT to a CBCT, the gas volumes as visible on that CBCT were delineated and assigned a density override of 0.01 in the deformed pCT, yielding a deformed CT [Figs. 1(d) and 1(f)] with the daily anatomical features (body contour, bony anatomy, gas volumes, and target position) as visible on CBCT [Figs. 1(c) and 1(e)].

Using the clinical plan, we calculated the dose distribution for the pCT (for the 4D‐CTs on the average scan) and each of the CBCTs (i.e., deformed pCTs) in Oncentra (Oncentra 4.3, Elekta, Stockholm, Sweden) for the clinically used patient position (i.e., the online fiducial marker‐based image registration). To investigate the effect of interfractional target position variation when fiducial markers would not be used, these calculations were also performed for a shift of the isocenter to simulate bony anatomy‐based position verification. For this, we used the bony anatomy‐based image registration that in the clinic is always performed prior to the fiducial marker‐based registration (XVI, version 4.5, Elekta, Sweden).

For dose accumulation, the fraction doses can be accumulated either deformably or rigidly. To use the deformable image registration to deform the dose distribution back to the planning CT, a voxel‐by‐voxel accuracy of the deformable image registration would be required.^19^ On CBCT, which suffers from poor soft tissue contrast and image artifacts, the target volumes as well as the organs at risk (OARs) are not well visible. Consequently, for these volumes, the deformable image registration is not accurate enough for dose accumulation. Therefore, we accumulated the dose rigidly using Velocity, exploiting the fact that for each patient all CBCTs had been rigidly registered to the pCT based on the fiducial markers. Thus, the fraction dose distributions were all in the same frame of reference as the pCT and could therefore be summed. Such rigid dose accumulation implicitly assumes all volumes remain in a fixed configuration with respect to the intratumoral fiducial markers. For the target volumes, this is a reasonable approach, as marker migration is minimal or absent.^10^ For the OARs we do not have such an independent measure of position; hence, rigid dose accumulation will likely not yield a reliable estimate of delivered OAR dose.

### 2.D Dose evaluation parameters

For each patient, we obtained DVHs in Velocity, for the planned dose distribution as well as for the two accumulated dose distributions (fiducial marker‐based and bony anatomy‐based). To account for differences in prescribed dose, dose distribution, and number of CBCTs between patients, we normalized the doses: for each patient, the planned dose DVH was scaled such that for the PTV D_98%_ = 95% (i.e., 98% of the PTV volume receives 95% of the prescribed dose);^20^ this factor was then applied to all accumulated dose DVHs of that patient as well.

As the iCTV‐to‐PTV margin also accounts for delineation uncertainties, using the iCTV to assess target dose coverage would be inadequate. To evaluate dose coverage, we therefore determined the mean dose (D_mean_), near maximum dose (D_2%_, highest dose to at least 2% of the volume), and near minimum dose (D_98%_) not only for the PTV but also for the iCTV_+5 mm_, defined as the iCTV expanded with a 5 mm margin (based on a margin = 2.5 Σ,^21^ with an estimated systematic error Σ of 2 mm due to delineation uncertainties).

For the surrounding OARs, rigid dose accumulation will not yield the actual delivered dose per voxel. However, as a coarse measure for (changes in) dose to surrounding healthy tissues, we did analyze the rigidly accumulated dose and determined D_mean_ and D_2 cc_ (highest dose to at least 2 cm^3^ of the volume) for the nearest OARs, i.e., duodenum and stomach.

For each DVH parameter, we tested the difference in group mean over the nine patients between planned and accumulated dose, for fiducial marker‐based and bony anatomy‐based image registration (two‐sided Wilcoxon signed‐rank test; *α* = 0.05). All analyses were done in R.^22^

### 2.E Parameters of anatomical change

We determined for each CBCT the interfractional tumor displacement, defined as the difference in table shift as obtained from the fiducial marker‐based and the bony anatomy‐based image registration. Per patient, we calculated the mean and range of the displacements in right–left (RL), posterior–anterior (PA), and superior–inferior (SI) directions.

To gauge the extent of the changes in gastrointestinal gas, we determined for each pCT and CBCT the relevant gas volume, i.e., the overlap between the gastrointestinal gas and the irradiated volume; as irradiated volume we selected the volume encompassed by the 20% isodose surface. For the CBCTs, this was done for the fiducial marker‐based registration only. We used a two‐sided Wilcoxon signed‐rank test to determine for each patient whether there was a difference in relevant gas volume between CBCTs and pCT (significance level *α* = 0.05).

## 3 RESULTS

The interfractional position variation varied considerably between patients (Table 1). The largest mean displacements were found in the SI direction, with a mean displacement of 6.5 mm in inferior direction for patient 2 and of 6.0 mm in superior direction for patient 3.

**Table 1:** Patient characteristics, including interfractional target displacement, internal clinical target volume, and relevant gas volumes

Patient	pCT	No. of CBCTs	Interfractional displacement [mm]^a^						iCTV volume [cm^3^]	Relevant gas volume [cm^3^]^b^		
			RL		PA		SI			pCT	CBCT	
			Mean	Range	Mean	Range	Mean	Range			Mean	Range
P1	3D‐CT	15	0.1	−3.8–3.3	−2.4	−7.4–5.0	−4.1	−14.6–3.3	207	154	51***	6–171
P2	4D‐CT	15	−0.9	−8.2–6.8	−0.1	−5.9–3.8	6.5	−2.2–18.8	47	73	56	13–143
P3	4D‐CT	15	2.9	−0.8–6.8	1.2	−1.3–2.9	−6.0	−12.3–−3.0	62	119	61**	5–132
P4	4D‐CT	15	−2.2	−9.1–4.9	−3.8	−13.9–1.2	−1.0	−8.6–8.3	150	111	53***	20–99
P5	4D‐CT	14^c^	2.2	0.1–4.3	0.9	−1.6–4.1	0.8	−5.2–6.6	57	69	63	18–155
P6	4D‐CT	15	−2.0	−9.2–3.4	−4.3	−6.8–0.0	−2.8	−8.2–1.4	149	60	65	15–150
P7	4D‐CT	13	0.9	−2.6–3.8	1.6	−1.0–4.0	4.0	0.0–6.6	103	394	212*	77–669
P8	4D‐CT	15	−2.7	−7.1–5.2	−3.9	−10.9–−0.1	2.7	−1.6–10.6	197	273	161**	21–503
P9	4D‐CT	15	0.6	−3.1–8.2	−2.3	−9.6–0.6	−1.0	−10.0–2.9	184	101	55***	21–97
Average^d^		14.7	−0.1 (SD 2.0)		−1.5 (SD 2.4)		−0.1 (SD 4.0)		128	150	86	

pCT, planning computed tomography; CBCT, cone‐beam computed tomography; iCTV, internal clinical target volume; RL, right–left; PA, posterior–anterior; SI, superior–inferior; SD, standard deviation.

a Difference in table shift between marker‐based and bony anatomy‐based image registration; means and ranges over the analyzed CBCTs; positive values indicate target volume displacement relative to bony anatomy in left, anterior, or inferior direction.

b Relevant gas volume = volume 20% isodose surface ∩ total gastrointestinal gas volume; for the CBCTs, this is the 20% isodose for marker‐based image registration. Significance of difference in relevant gas between CBCTs and refCT (two‐sided Wilcoxon rank‐sum test): *P < 0.05; **P < 0.01; ***P < 0.001.

c For one CBCT, artifacts prevented reliable image registration.

d Average is the mean over (the means of) the nine patients; SD is SD of the nine means.

**Figure 2:**
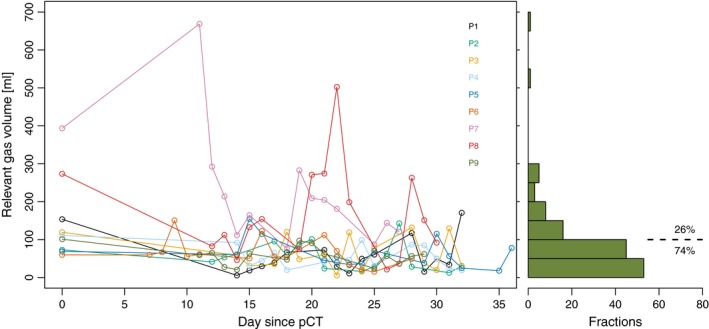
Absolute relevant gas volume. For each of the nine patients, the relevant gas volume on pCT and cone‐beam CT (i.e., volume of gastrointestinal gas within the 20% isodose surface) in cm^3^ over the course of treatment. The histogram shows the distribution for all 132 fractions (pCT data excluded); for 74% (98/132) of fractions, the relevant gas volume is <100 cm^3^.

The mean relevant gas volume on pCT was 150 cm^3^ (Table 1). Over all CBCTs, the range of relevant gas volume was 5–669 cm^3^, with for 74% (98/132) of CBCTs a relevant gas volume <100 cm^3^ (Fig. 2). For 83% of CBCTs, the relevant gas volume was smaller than the relevant gas volume on the respective pCT. Shape, position, and volume of gastrointestinal gas could vary considerably between fractions, as illustrated in Fig. 1, which shows for patient 8 the gas volume on pCT [237 cm^3^; Fig. 1(b)] and on CBCT for fraction 9 [503 cm^3^; Fig. 1(d)] and fraction 11 [21 cm^3^; Fig. 1(f)]. For six out of nine patients, the relevant gas volume differed significantly between CBCTs and pCT (Table 1); for these six patients, the mean relevant gas volume was smaller on CBCT than on pCT.

**Figure 3:**
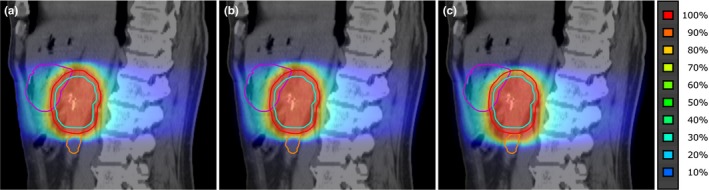
Planned and accumulated dose distributions. Colorwashes for (a) planned dose and accumulated dose for (b) fiducial marker‐based position verification and (c) bony anatomy‐based position verification for patient 3 (projected onto a sagittal view of the pCT). Contours are shown for iCTV_+5 mm_ (light blue), planning target volume (PTV; red), stomach (purple), and duodenum (orange). Two fiducial markers are visible within the iCTV_+5 mm_.

For patient position verification based on fiducial markers, the accumulated dose distribution differed very little from the planned distribution (Table 2). Figures 3(a) and 3(b) show these distributions for a typical example (patient 3). For bony anatomy‐based registration, the accumulated dose distribution [Fig. 3(c)] could be quite different than the planned dose distribution [Fig. 3(a)]. Due to daily differences in position of the tumor (fiducial markers) relative to the bony anatomy, the high‐dose volume is smeared out and shifted for bony anatomy‐based dose accumulation [Fig. 3(c)]. For the average D_98%_ of the PTV, there was a significant difference (*P* = 0.004) between the bony anatomy‐based accumulated dose (85.9%) and the planned dose (95.0%). D_98%_ of the iCTV_+5 mm_ dropped to <95% for patient 2 (from 95.9% to 85.8%) and patient 4 (from 97.2% to 94.7%). D_mean_ of the stomach differed significantly between the fiducial marker‐based accumulated dose and the planned dose; however, with a D_mean_ of 22.2% (planned) vs. 22.6%, this difference was not clinically relevant. For all other tested DVH parameters, differences were not significant (Table 2).

**Table 2:** DVH parameters presenting dose coverage and dose to the organs at risk closest to the target. D_98%_ > 95% for both position verification strategies and the difference is small (96.7% vs. 95.3%). For individual patients, however, the use of bony anatomy‐based position verification can yield considerable underdosage (up to D_98%_ = 85.8%)

Volume	DVH parameter	Planned dose		Accumulated dose			
				Marker‐based		Bony anatomy‐based	
		Mean [%]	Range [%]	Mean [%]	Range [%]	Mean [%]	Range [%]
iCTV_+5 mm_	D_98%_	96.3	95.5–97.8	96.7	96.4–97.0	95.3	85.8–97.9
	D_mean_	98.7	97.8–101.1	99.0	98.2–100.0	98.8	96.7–100.1
	D_2%_	100.9	99.2–104.5	101.2	99.7–103.3	100.6	98.8–102.4
PTV	D_98%_	95.0	95.0–95.0^a^	95.4	94.3–96.4	85.9*	72.1–92.9
	D_mean_	98.4	97.5–100.4	98.7	97.9–99.4	97.5	94.1–98.8
	D_2%_	100.8	99.3–104.3	101.2	99.7–103.1	100.6	98.7–102.3
Duodenum stomach	D_mean_	80.9	44.2–99.2	81.3	44.7–97.9	80.4	45.3–98.1
	D_2 cc_	99.9	96.5–103.2	100.0	96.0–101.9	99.6	97.0–101.4
	D_mean_	22.2	11.4–51.8	22.6*	11.4–52.4	23.3	14.6–55.6
	D_2 cc_	90.9	62.9–104.2	91.4	63.7–102.2	92.2	66.4–101.7

Difference in mean between planned and accumulated dose is tested (two‐sided Wilcoxon signed‐rank test); *P < 0.01.

DVH, dose–volume histogram; iCTV_+5 mm_, internal clinical target volume expanded with a 5 mm margin; PTV, planning target volume. Means and ranges over all nine patients.

a For each patient, *all* DVHs are scaled with a single factor such that for the *planned* dose distribution the D_98%_ of the PTV = 95%.

**Figure 4:**
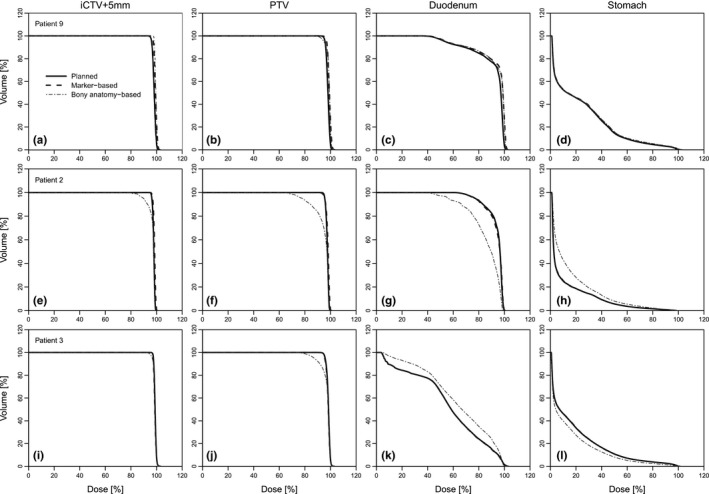
Dose–volume histograms (DVHs). For iCTV_+5 mm_, planning target volume (PTV), duodenum, and stomach, for patients 9 (a–d), 2 (e–h), and 3 (i–l), data are shown for planned dose (solid lines), accumulated dose for fiducial marker‐based position verification (dashed lines), and accumulated dose for bony anatomy‐based position verification (gray dotted lines).

Figure 4 shows DVHs for three patients, one (patient 9) with a small and two (patients 2 and 3) with a large mean difference between fiducial marker‐based and bony anatomy‐based positioning. For patient 9, there was little difference between planned and either accumulated dose DVH, for target [Figs. 4(a) and 4(b)] as well as for OARs [Figs. 4(c) and 4(d)]. For patients 2 and 3, the differences between planned and fiducial marker‐based accumulated dose were also very small. For the bony anatomy‐based accumulated dose, however, the DVHs differed considerably. For patient 2 (mean tumor displacement of 6.5 mm in inferior direction), the dose to the stomach increased [Fig. 4(h)] and the dose to the duodenum decreased [Fig. 4(g)]. For patient 3, the mean displacement was 6 mm in superior direction, yielding the opposite effect [Figs. 4(l) and 4(k)].

**Figure 5:**
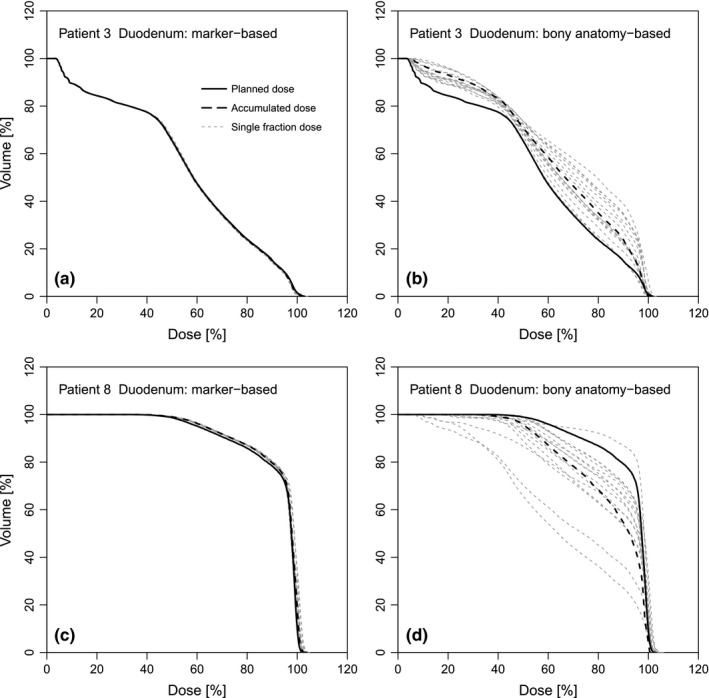
Fraction doses. Dose–volume histograms (DVHs) for the duodenum of patients 3 (a and b) and 8 (c and d). Data are shown for fiducial marker‐based position verification (left panels) and bony anatomy‐based position verification (right panels) for planned dose (solid line), accumulated dose (dashed line), and each of the 15 fraction doses (gray dashed lines).

**Figure 6:**
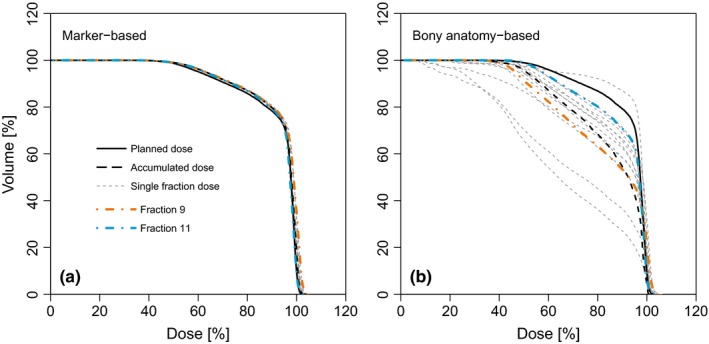
Fraction doses for small and large relevant gas volumes. For patient 8, dose–volume histograms (DVHs) for the duodenum, with data shown for (a) fiducial marker‐based position verification and (b) bony anatomy‐based position verification (same as Fig. 5). DVHs are highlighted for the two fractions depicted in Figs. 1d and 1f: fraction 9 (orange; relevant gas volume: 503 cm^3^) and fraction 11 (blue; relevant gas volume: 21 cm^3^).

For two patients (patients 3 and 8), we plotted the DVHs of the duodenum for the individual fractions, together with the DVHs for planned and accumulated dose distributions, for the fiducial marker‐based position verification [Figs. 5(a) and 5(c)] and bony anatomy‐based position verification [Figs. 5(b) and 5(d)]. For the fiducial marker‐based position verification, the DVH of each fraction closely resembled the DVHs of planned and accumulated dose, indicating a very limited effect of gas and body contour changes. This was even the case for the large differences in relevant gas volume seen in patient 8 (see Fig. 6, in which the DVHs are highlighted for the fractions with largest (503 cm^3^) and smallest (21 cm^3^) relevant gas volume, corresponding to the deformed CT images in Figs. 1(d) and 1(f), respectively). For the bony anatomy‐based position verification, on the other hand, the fraction DVHs differed considerably from each other [Figs. 5(b), 5(c), and 6(b)], demonstrating that the accumulated dose is largely affected by interfractional target position variation.

## 4 DISCUSSION

In this study, we investigated the dosimetric effects of anatomical changes for fractionated radiation therapy in the upper abdomen, by retrospectively calculating and accumulating daily dose using the CBCTs from 3‐week fractionated treatments of nine pancreatic cancer patients. The robustness of dose coverage was analyzed using an especially for this purpose defined volume, the iCTV expanded with a 5 mm margin. For position verification based on intratumoral fiducial markers, target dose coverage was little affected, signifying the limited dosimetric effect of changes in gastrointestinal gas and body contour for fractionated photon radiation therapy. Dose accumulation based on bony anatomy‐based position verification, however, showed that interfractional target position variations can decrease target dose coverage considerably.

To analyze target dose coverage, others have used the PTV^11^ or the CTV,^12^ both of which are not the most appropriate. The PTV includes a margin applied to ensure CTV dose coverage while accounting for (geometrical) uncertainties. To evaluate the effect of said uncertainties by analyzing the dose to the PTV will therefore overestimate dosimetric effects. On the other hand, using the CTV will underestimate changes in target dose coverage, as part of the CTV‐to‐PTV margin accounts for delineation uncertainties. For pancreatic cancer, the observer variation in target delineation can be considerable;^23^, ^24^, ^25^ e.g., one study observed a mean difference in center of mass between twice delineated target volumes of 3.0 mm,^23^ while another found a mean overall observer variation of 8.0 mm for the delineated iGTV for four patients.^24^ However, studies are often limited in size and report different parameters and, in addition, the observer variation is not uniform over the surface of the volume. Therefore, in our study, we used the iCTV_+5 mm_ volume to report target dose coverage, with the margin of 5 mm based on a (low) estimate of the delineation uncertainty (systematic error Σ) of 2 mm.^21^

The limited dosimetric effects of changes in gas and body contour for photon irradiation have been previously reported by others.^3^, ^4^, ^6^ For prostate cancer VMAT plans, the effect of body contour changes had been investigated by simulating body contour increases/decreases in lateral and anterior directions.^3^, ^4^ To calculate daily dose distributions in our study, we used deformably registered reference CTs with gastrointestinal gas volumes copied from CBCT, which represented the daily body contour, bony anatomy, target location, and gas as visible on daily CBCT. By accumulating dose for both bony anatomy‐based and fiducial marker‐based position verification, we were able to distinguish between the effects of interfractional target position variation and the effects of changes in gas and body contour. For each patient, we used all 13–15 available CBCTs, ensuring our accumulated dose distributions were representative for 3‐week fractionated radiation therapy courses.

When looking at the group mean, dose coverage appears sufficient (i.e., D_98%_>95%; Table 2), even for bony anatomy‐based position verification. However, the group mean is not the appropriate metric to evaluate when assessing target dose coverage; for a clinical relevant evaluation, considering the worst case is more suitable. As results differed considerably between patients, the nine patients included in our study may not be sufficient to fully assess the implications of anatomical changes on target dose coverage for individual patients.

Another limitation in our study was the low soft tissue contrast of CBCT; as shown for the prostate,^26^ this prevents accurate deformable image registration for soft tissues, such as pancreas and surrounding organs. For calculated overall dose distributions, the effect of inaccurate soft tissue registration is very limited due to the limited difference in HU between the different soft tissues. For dose accumulation, however, inaccurate registration of organs may greatly affect the accumulated dose within an organ. Therefore, we chose to accumulate the dose rigidly, which implicitly assumed all structures to move rigidly with the fiducial markers. For OARs, this is evidently not the case and DVHs only provide an indication of (changes in) dose to healthy tissues, but cannot be used to report the actual delivered dose to a specific organ. For the target volume, though, the intratumoral fiducial markers indicate its position, as migration has been found to be minimal or absent.^10^ Thus, even though rotations and deformations are not taken into account, rigidly accumulated dose can be used as a valid measure for target dose coverage. However, daily imaging that also visualizes target volume deformations (e.g., MRI or CT) may enable more accurate dose accumulation.

A consequence of the application of fixed CTV and PTV margins and direct copying of the gastrointestinal gas volumes from CBCT to the deformed CT is that the delineated gas can overlap with the iCTV and/or PTV. As gas will result in a decreased calculated dose in that part of the iCTV/PTV, a change in overlap possibly affects target dose coverage for that fraction. For 110 of the 132 fractions (83%), the overlap of gas and iCTV_+5 mm_ was <1% of the iCTV_+5 mm_ volume (fiducial marker‐based position verification). The mean overlap over the fractions was ≤0.5% for seven out of nine patients; for patients 2 and 6 the mean overlap was 0.8% and 1.7%, respectively. With the overlap being small, and not for every fraction at the same location, the overall effect of the overlap on the accumulated dose can be expected to be minimal, as can also be inferred from the limited discrepancies between planned and marker‐based accumulated dose for iCTV and PTV. Applying a density override of 1 to the overlap may partially circumvent this issue.

The relevant gas volume was smaller on CBCT than on pCT for the majority of CBCTs. Whether this is a consequence of the radiation therapy is unknown; no apparent time trends in volume were observed (Fig. 2). In particle therapy, changes in gastrointestinal gas can severely affect target dose coverage, as established for carbon ion and proton therapy in earlier studies of our group.^17^, ^27^ The observed limited effects of changes in gastrointestinal gas in our current study illustrate the inherent robustness of photon radiation therapy.

With the use of an iCTV or ITV, respiration‐induced motion is incorporated into this internal target volume and not into the CTV‐to‐PTV margin. *Changes* in respiration‐induced motion during the course of radiotherapy, which are likely to take place,^28^ can affect target dose coverage; an increase in motion with respect to the planning CT may compromise target coverage. Intrafractional motion, however, is outside the scope of our current study, in which we focused on the effects of interfractional changes in gastrointestinal gas, body contour and target position.

In our study, the CTV‐to‐PTV margin was 10 mm. This margin is used clinically for the fiducial marker‐based daily online position verification, but may also be used for bony anatomy‐based position verification.^15^ Ideally, the applied margin is determined based on calculations incorporating quantified uncertainties determined specifically for the patient population and applied treatment technique. In practice, however, this is not always the case, due to a lack of data on the various uncertainties or the choice made by the clinician in the tradeoff between target dose coverage and dose to healthy tissue. The decrease in target dose coverage for large interfractional position variations when using bony anatomy‐based position verification could unquestionably be remedied by the use of a CTV‐to‐PTV margin larger than 10 mm. This, however, would also increase dose to surrounding organs, especially as the required margins may be >20 mm when taking measured interfractional displacements^10^ and delineation uncertainties^24^ into account. Therefore, the use of fiducial marker‐based position verification is preferred for pancreatic cancer patients.

## 5 CONCLUSION

Fractionated photon irradiation of pancreatic tumors is robust against changes in body contour and gastrointestinal gas, with dose coverage only mildly affected. Interfractional tumor position variation, however, can greatly affect target dose coverage when using bony anatomy‐based position verification. To ensure target coverage and minimize dose to surrounding healthy tissues, intratumoral fiducial markers and daily online position verification are imperative.

## CONFLICT OF INTEREST

The authors declare that they have no conflicts of interest.
